# Patients’ Perspective of Medication Safety in Hungary: A Netnography-Based Mixed-Method Content Analysis

**DOI:** 10.3390/healthcare14030397

**Published:** 2026-02-04

**Authors:** Barbara Báldy, Judit Lám

**Affiliations:** 1 Doctoral College, Mental Health Sciences Division, Interdisciplinary Social Sciences Doctoral Program, Semmelweis University, 1085 Budapest, Hungary; 2 Health Services Management Training Centre, National Laboratory for Health Security, Data-Driven Health Division, Semmelweis University, 1125 Budapest, Hungary; lam.judit@emk.semmelweis.hu

**Keywords:** medication safety, patient safety, patient perspective, netnography, mixed-method content analysis

## Abstract

**Highlights:**

**What are the main findings?**

- A netnography-based mixed-method content analysis of Hungarian online patient discussions (5174 comments, 2020–2023) revealed that medication safety concerns are predominantly centered on issues related to access to healthcare and communication between patients and providers. Patient-reported concerns regarding medication safety are primarily motivated by barriers to access and ineffective communication; other safety domains at the system level are minimally represented in online discourse.
- Online discussions regarding medication safety are predominantly led by patients, with limited involvement from healthcare professionals, thereby exposing a notable deficiency in professional guidance and presence within the digital environment. Medication safety concerns are concentrated within specific therapeutic areas, notably gynecology and gastroenterology. These concerns are closely aligned with corresponding ATC main groups, thereby facilitating targeted, patient-centered interventions.

**What are the implications of the main findings?**

- A netnography-based mixed-method content analysis has demonstrated itself as a practical and scalable approach for identifying real-world patient safety signals, providing actionable insights to guide medication safety strategies and patient engagement initiatives.
- Empowering patients as active partners in medication safety (e.g., through better education, error reporting, and feedback) can help prevent errors and improve patient safety outcomes.

**Abstract:**

**Background/Objectives**: Medication-related safety incidents rank among the most prevalent patient safety concerns globally. In addition to healthcare professionals, patients also play a vital role in ensuring safe medication practices. To effectively engage them, it is essential to gain a deeper understanding of their knowledge and perspectives. **Methods**: We conducted a netnography-based mixed-method content analysis study within the Hungarian online environment to identify key patient concerns. A total of 5174 relevant comments and discussions were analyzed (from 14 August 2020 to 14 August 2023), utilizing a medication safety framework based on Glies et al. The analysis was confined to publicly accessible online content related to oral medications and did not include demographic information about commenters. **Results**: The framework was applicable, though its representation was uneven. Patients predominantly focused on issues related to Access to services and Communication. Online discussions were primarily dominated by patients, with contributions from relatives and healthcare professionals being comparatively limited. The majority of concerns pertained to prescription medications, particularly in the fields of gynecology, internal medicine, and gastroenterology. ATC codes G and A were most frequently referenced, corresponding to the healthcare domains discussed. **Conclusions**: Initiatives aimed at enhancing medication safety should prioritize improving access and communication. Patients must be empowered as active agents in safety efforts; they can aid in preventing errors, reporting incidents, and offering feedback. Their engagement supports organizational learning and promotes safer healthcare delivery.

## 1. Introduction

Medication management has become a widely discussed topic, particularly in light of increased trends in medication use and the evolving roles of patients [1]. Patients play a central role in medication management and seek to acquire more information regarding their medication, particularly those with high health literacy [2]. Furthermore, medication-related issues are the most significant concern in patient safety [2]. Nevertheless, our understanding of patients’ perspectives, barriers, and strategies remains incomplete, as many researchers primarily focus on healthcare professionals discussing patients. Therefore, there is a necessity to also consider the patients themselves [2].

Furthermore, we lack information regarding Hungarian patents in this area. While numerous publications utilize survey instruments or traditional qualitative research methodologies, there is no available data concerning online sources employed to investigate this subject. Online discussions provide genuine insights into patients’ mindsets and perspectives. Our overarching research objective is to gain a deeper understanding of patients’ views on medication safety in order to develop educational tools aimed at enhancing their safety.

According to WHO data, the cost of medication-related harm amounts to approximately US$ 42 billion annually worldwide [3]. In high-income countries, medication errors occur in approximately 2–3% of primary care cases, and in hospital settings, 10% of harm cases are attributable to medications, which represent the most common source of adverse events [4]. The World Health Organization’s medication safety strategic framework advocates for the establishment of four primary subdomains within which targeted actions can be undertaken to prevent errors: public awareness and medication literacy, patient engagement, reporting by patients, and the involvement of patient organizations [3]. Regarding medication safety, it is understood that a lack of communication may constitute the most common issue from the perspective of patients [5]. Patients report inadequate communication with their healthcare professionals as the most significant factor affecting their safety [5]. Effective communication is essential to actively involving patients in their own care [6]. Another issue could concern the factors related to the patient and their relatives, such as patient knowledge, patient responsibility, physical and cognitive considerations, and the degree of patient involvement [5]. Numerous patients report experiencing a deficiency in information while concurrently perceiving their understanding of their medications as sufficient [5]. In the context of intravenous infusions, however, patients’ knowledge appears to vary more in both depth and scope [7]. Additionally, they encounter numerous obstacles in assuming responsibility, such as adapting to a new medical routine [5]. Regarding the dosing process, patients are knowledgeable about certain aspects; for instance, they are more aware of changes in medication color or name, but demonstrate less awareness or concern regarding the correct dosage or appropriate route of administration within hospital settings [6].

It is important to highlight the significance of healthcare professionals, as patients may be apprehensive if these professionals do not consider potential drug interactions or demonstrate a lack of knowledge in this domain [5]. Other safety issues may emerge from the inadequate design of electronic systems, potentially resulting in errors or missed doses, as patients might encounter difficulties in accessing their medication promptly (for example, when prescriptions are automated and not updated in the system in a timely manner) [5]. If patients miss their regular (original) medication, the use of generic products might cause confusion and could contribute to medication errors [5]. Patients demonstrate a willingness to self-administer their medications within hospital settings and express appreciation when healthcare providers support this practice [8]. This attitude may contribute to increased adherence and improved collaboration between healthcare professionals and patients, particularly in the context of self-administration of oral and non-parenteral medicines, and may support correct medication use following discharge [8,9].

The concept of utilizing virtual spaces for ethnographic research is increasingly gaining popularity, as researchers can observe and document the behaviors of communities and cultures, akin to traditional field studies in ethnography [10]. Online communication among consumers has been examined through netnography to comprehend their attitudes, perceptions, and emotions [11]. Netnography constitutes an innovative qualitative research methodology that enables the examination of groups of individuals sharing common interests and their collective information [10]. These data are typically text-based; however, alternative material types such as audiovisual presentations, images, and photographs can also be encountered [10]. Patients are progressively resorting to the internet when seeking information or alternative communities that share similar issues [11]. Medication can be a sensitive topic, and obtaining authentic information on such matters is often challenging; however, with the application of netnography, we posit that it is possible to observe the patients’ perspectives. Netnography is primarily a qualitative research method; in this study, it is utilized as a component of a mixed-method content analysis.

Based on the previous research findings, our research questions are as follows:

Methodological Validation

1. Is netnography an appropriate methodology for exploring patient perspectives related to medication safety within health research?
2. Can netnographic studies carried out within the Hungarian online environment substantiate the patient-centered medication safety framework proposed by Giles et al. [5]?

Participation and Visibility in the Online Environment

3. Who constitute the primary participants engaged in online discussions concerning medication safety?

Patterns Related to Medication and Context

4. Which categories of oral medications, medication groups (ATC codes), and healthcare sectors are most commonly associated with discussions on medication safety?

Patient-Reported Safety Concerns

5. What are the principal medication safety issues highlighted by patients in online communication?

## 2. Materials and Methods

The analytical workflow adhered to a sequential process comprising data retrieval, query refinement, manual relevance screening, framework-based coding, and final tagging.

Data was collected over a three-year period, from 14 August 2020 to 14 August 2023. The dataset includes archival data, which pertains to communications and postings made by online members prior to the researcher’s entry into the online space, yet remains accessible for review. Additionally, data were generated through reflexive field notes documented by the researchers [10].

In this study, we employed SentiOne (Automate version 224, Poland), owing to its provision of the most comprehensive data within the publicly accessible Hungarian online environment. To identify pertinent data, keyword searches were conducted. The keywords utilized included: tablet, capsule, syrup, suspension, along with ninety-three exclusion keywords aimed at refining the data clarity (e.g., child, tourism, cosmetics). A complete list of both inclusion and exclusion keywords is detailed in Appendix A.

This study concentrates on oral medications due to their safety being predominantly reliant on active patient participation and generally administered independently outside direct healthcare supervision. Oral medications are the most prevalent form of treatment in routine outpatient care and necessitate patients to oversee dosing, timing, adherence, and potential interactions independently, thereby making them particularly pertinent for examining medication safety within real-world settings.

Owing to the intricacy of the keyword network, data collection was performed utilizing advanced queries within the SentiOne platform. During the query construction phase, the dataset was persistently monitored through numerous test queries, each succeeded by data exportation and manual examination. Based on these iterative assessments, supplementary exclusion keywords were incorporated until further refinement was impractical without compromising the retention of pertinent content.

Following the final query, relevant items were manually selected and subsequently coded. Content was considered relevant if it contained references to medication use, medication names, and/or related experiences, opinions, or suggestions expressed by commenters. Duplicate entries were removed from the dataset, and short comments were retained only if they meaningfully addressed the above criteria.

The archived data accurately represent the state of the content at the time of collection. Post-publication edits or deletions of comments were not traceable and therefore could not be included in the analysis. Temporal variation and trend analysis were intentionally omitted from the study design, as the primary objective was thematic exploration rather than longitudinal assessment.

We compiled the pertinent discussions in an Excel (Office 2019, USA) spreadsheet and assigned appropriate labels. The primary labels reflected the issues within the frameworks, and a single comment could be associated with two framework labels if it addressed two areas. To determine the most suitable framework label for each comment, we employed the respective definitions (Table 1). The data were initially coded by one researcher and subsequently reviewed in full by a second researcher. In cases of disagreement, the original framework definitions were revisited and discussed until consensus was reached on the most appropriate category for the given comment. In Appendix A, we provide illustrative examples for each category based on the relevant data.

Quantitative analysis was confined to descriptive frequency counts. The coding scheme permitted variation across analytical dimensions: in certain categories, each comment could be assigned only one label, while in others, dual coding was allowed. Percentages are therefore presented within each coding dimension and sum to 100%. This distinction—whether single or dual labels could be assigned—is explicitly indicated in the corresponding tables. Percentages are reported in rounded form and are utilized solely for descriptive purposes, to support qualitative interpretation rather than to convey statistical precision.

In addition to the framework tags, we classified our data according to the commenter’s identity (patient, relative, healthcare professional, or other) and the relevant healthcare sector (such as gynaecology, internal medicine, or “unspecified [non-specific symptoms]” when the comment described general symptoms—e.g., headache, common cold—without a specific healthcare specialty being identifiable). The categorization was based on the content of the comment, considering the disease mentioned, the medicinal product referenced, the type of oral medication (prescription, over-the-counter, herbal), and ATC codes. We employed the World Health Organization’s classification system, tagging only the first level of the fourteen main anatomical or pharmacological groups [12]. Main ATC groups were assigned when a medicine could be clearly identified; otherwise, the comment was categorized as “not identifiable (ATC).”

With regard to the members involved in the discussions, we lack information concerning their demographic status.

The ethical license number for the research is BM/15379-1/2023, issued by the Medical Research Council of the Hungarian Ministry of Interior.

## 3. Results

The initial query identified a total of 122,772 comments, of which 5174 were deemed relevant. The process of data cleaning was employed to determine the relevant comments. The majority of these comments originated from health forums and public Facebook pages. Specifically, 2011 comments were sourced from Facebook, with the remaining contributions collected from online forums and various other websites.

Inferential statistical analyses were not employed, given that the principal objective of the study was to undertake a thematic exploration of patient-reported experiences. The dataset was obtained from non-probabilistic, naturally occurring online content, wherein individual observations are not independent; thus, population-level inference would be methodologically inappropriate. Consequently, the analysis was concentrated on descriptive frequencies and proportions to facilitate a transparent presentation of predominant themes. All reported percentages denote within-dimension normalized proportions, calculated relative to the total number of coded instances within the respective analytical category.

### 3.1. The Framework

At least one comment was identified for each framework category; however, the distribution across categories was uneven. The highest frequencies were observed in the ‘Access to services’ and ‘Communication’ domains, which together accounted for the majority of medication safety-related comments (Table 2).

Within the ‘Access to services’ category, two recurring types of difficulties were reported. First, patients described challenges in reaching their physicians when questions regarding medication use arose. Second, patients with complex medication-related concerns—often involving consultations with multiple specialists—reported seeking information online after failing to obtain clear or satisfactory answers through formal healthcare encounters.

In the category of ‘Communication,’ comments predominantly detailed interactions with healthcare professionals. Patients often recounted experiences characterized by dismissiveness, impersonality, or discourtesy, and noted the limited opportunities available for inquiring or voicing concerns during consultations. Other categories within the framework were less prevalent in the dataset. Notably, ‘Patient- and carer/relative-related factors’ and ‘Dignity and respect’ were represented by a comparatively small number of comments.

Having outlined the distribution of medication safety issues across framework categories, the subsequent section assesses the participants involved in these online discussions.

### 3.2. The Commenters

The data indicates that the majority of comments were contributed by patients; other contributors were less engaged in the discussions. This underscores that medication safety is a significant topic for patients who have experienced or are experiencing medication safety issues. However, relatives and healthcare professionals are not as actively involved as the patients themselves (Table 3).

Beyond identifying the primary contributors to the discussions, the subsequent analysis concentrates on the categories of oral medications most commonly linked with patient-reported safety concerns.

### 3.3. The Type of Oral Medications

The majority of the comments were related to RX (medical prescription) medications, indicating that patients encountered the most safety concerns with these types of drugs. Significantly fewer questions and issues were associated with over-the-counter (OTC) medications and herbal remedies (Table 4).

To further contextualize these healthcare domains, the subsequent analysis associates reported concerns with the main groups of the Anatomical Therapeutic Chemical (ATC) classification system.

### 3.4. The Healthcare Areas

Concerning the healthcare domains, the data exhibited variability. The most frequently referenced area was gynecology, with the majority of comments related to reproductive medicine. The second-most-mentioned fields were general medicine, internal medicine, and gastroenterology, which was anticipated due to their impact on a significant portion of the patient population (Table 5).

### 3.5. The ATC Codes

Based on the ATC codes, the ‘G code’ was the most frequently mentioned, with these comments predominantly associated with the gynecology field (1235 comments out of a total of 1328). The ‘A code’ was the second-most-mentioned ATC code, most often linked to gastroenterology (473 comments), internal medicine (250 comments), and the general medical field (136 comments). The remaining ATC codes were less frequently referenced; however, it can be observed that the medical specialties correspond well with the respective ATC codes (Table 6).

## 4. Discussion

In our research, we examined patients’ perspectives on medication safety utilizing a netnography-based mixed-method content analysis research methodology. To systematically organize our data and gain deeper insights into the subject, we employed a pre-existing framework founded on Giles et al. [5], and we classified the data with additional categories: commenters, type of oral medication, healthcare area, and ATC codes. Relative to the total volume of raw data, the relevant comments were significantly fewer (5174 relevant comments out of 122,772), due to the inability to utilize ‘medication safety’ or ‘patient safety’ as keywords. These terms are exclusively employed in articles by journalists and scientists. Regrettably, in the Hungarian language, the chosen keywords possess multiple meanings, which prolonged the data cleaning process.

**Figure 1:**
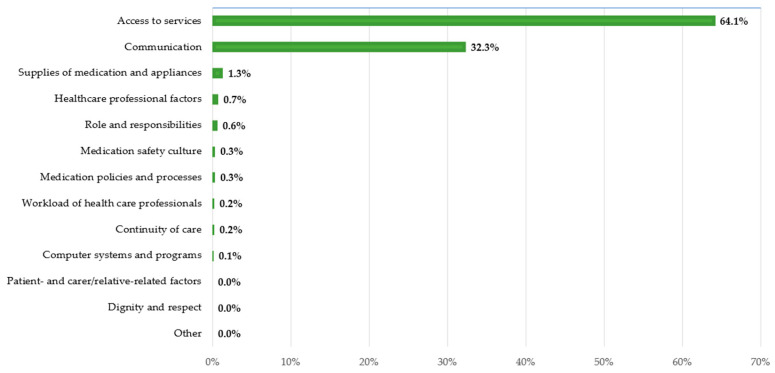
‘Access to services’ and ‘Communication’ emerged as the most dominant categories in the online discussions.

Within the Hungarian population, the framework was effective and proved to be valuable; however, the items within the framework were disproportionately distributed. The most significant aspects identified were ‘Access to services’ and ‘Communication’, wherein patients engaged in discussions and encountered the greatest challenges, particularly concerning medication safety (Figure 1). These results are consistent with previous research [5], and the results of the IPSOS Global Health Service Monitor highlight that access to treatment and waiting times constitute a problem for 42% of the global population, with Hungary experiencing the most significant issue at 65% [13]. Regarding other topics, concerning the mechanism of the healthcare system, patients exhibit limited awareness. The infrequent occurrence of comments concerning ‘Patient- and carer/relative-related factors’ may suggest a limited level of patient self-reflection regarding personal roles and responsibilities in medication safety; however, alternative explanations cannot be entirely discounted. Likewise, the limited representation of the ‘Dignity and respect’ category may be associated with the tendency of online discourse to less frequently explicitly describe positive or affirming care experiences. This observation aligns with the broader characteristics of Hungarian online conversation, where negative experiences are more frequently expressed than positive ones. Therefore, a low visibility of specific framework domains within online discussions should not be interpreted as their insignificance in clinical practice but rather as a limitation of patient-generated digital data, which underscores the necessity for supplementary research methodologies.

Access to healthcare significantly influences life expectancy, particularly among low-income populations and residents of rural areas [14], and in Hungary, over the past decade, the private healthcare sector has experienced a growing tendency, attributable to the deficiencies within the public healthcare sector [15]. Pharmacists possess the professional competence to assist patients and their relatives in numerous aspects related to medication safety; however, this role is not yet broadly acknowledged or routinely employed at the societal level [16]. Pharmacies are broadly accessible healthcare facilities where pharmacists are available to offer medication counseling, clarify dosing instructions, manage common adverse effects, and promote safe medication use. The patient expresses concern that this readily available resource remains underrecognized and underutilized. This disparity indicates that strategies for medication safety should not solely concentrate on expanding services but also on enhancing patient awareness of existing support systems and elucidating the role of pharmacists in managing daily medication safety issues, especially in a setting where numerous patients face growing challenges in accessing healthcare. Communication continues to be a recognized challenge: numerous studies have identified insufficient information as a significant problem and deficiency—consequently, the importance of effective information transfer has become more evident [17,18,19,20]. The World Health Organization’s medication safety strategic framework also emphasizes that public awareness and medication literacy are critical factors in the prevention of errors [3]. Our findings reaffirm that communication between patients and healthcare professionals remains a significant issue, and patients continue to have unmet needs concerning medication safety. Effective communication is essential, as accurate information from patients can help prevent errors.

Patients who lack access to healthcare services and are compelled to seek solutions for their medical safety concerns through online platforms are particularly affected. However, within a traditional in-person healthcare system, family members could play an important role; in the digital space, their involvement is less likely. Furthermore, healthcare professionals have a disproportionately low visibility in online environments, which could result in misinformation if patients exchange advice among themselves without guidance from qualified professionals.

The increasing utilization of artificial intelligence, especially large language models (LLMs), possesses the capacity to significantly revolutionize online health communication, including patient discussions concerning medication safety. The following discussion of artificial intelligence is intended to outline potential future implications rather than to report findings derived directly from the present analysis. AI-based tools have the potential to assist patients by delivering timely, structured, and comprehensible information, thereby aiding clarifying medication use, potential adverse effects, and common safety concerns when access to healthcare professionals is constrained. Nonetheless, these technologies also present significant risks. In the absence of proper governance, validation, and professional oversight, responses generated by AI may disseminate incomplete, outdated, or misleading information, which could amplify safety risks rather than mitigate them. Consequently, the integration of AI into online patient communication platforms should emphasize reliability, transparency, and adherence to evidence-based clinical guidelines. Furthermore, such integration should be incorporated within broader strategies for patient safety and health literacy, rather than functioning as an isolated solution.

The most critical domain was gynecology, particularly concerning G ATC code medications, such as reproductive drugs. It is presumed that the majority of respondents were women. Evidently, women appear to be more engaged in issues related to medication safety, a finding that aligns with prior research in the field of patient safety [21,22,23]. The second-most-frequently mentioned areas were in the general field, internal medicine, and gastroenterology, which was not surprising because these areas impact the majority of the patient population. Although cardiovascular diseases constitute one of the leading causes of morbidity and mortality in Hungary [24], concerns regarding the safety of cardiology-related medications were observed to arise relatively infrequently within online patient discussions. This discrepancy may reflect differences in the articulation of medication safety issues across diverse patient groups rather than indicating a lower level of clinical risk. One plausible explanation is that patients with chronic cardiovascular conditions are more frequently enrolled in stable, long-term care pathways characterized by established medication routines and regular follow-up, which may diminish the perceived necessity for online information-seeking. Alternatively, the reduced visibility of cardiology-related concerns could be attributed to decreased digital engagement among older patient populations, who are disproportionately affected by cardiovascular diseases, or to the normalization of long-term medication use in daily life. Importantly, the limited occurrence of cardiology-related medication safety concerns in online discourse should not be misconstrued as evidence of lower medication-related risk. Instead, it may highlight a potential blind spot in patient-reported safety signals, emphasizing the importance of utilizing additional data sources to adequately capture safety concerns in populations that are less represented in digital environments.

Overall, netnography serves as a valuable instrument for gaining deeper insights into the patient’s perspective. These findings can assist in identifying areas where efforts should be directed towards patient outreach and education concerning medication safety. Throughout various topics, the commenters posed numerous questions, indicating a strong willingness among most individuals to further understand medication safety and to engage more actively, particularly the patients themselves.

These results imply that healthcare systems could profit from paying increased attention to these needs when aiming to support patients in enhancing their medication management safety. Although this study is grounded in the Hungarian healthcare context, the identified patterns of patient experiences and medication safety concerns are not context-specific. By applying a netnographic approach to publicly accessible online discussions, this study demonstrates the broader relevance of patient-generated data for understanding everyday medication safety challenges across different healthcare systems.

### Limitations

Our research is exclusively focused on oral medications; other types of medication have been deliberately excluded as the keywords are centered on oral administration. This predominant focus on oral medications constitutes a scope-related limitation of the study. While this approach aligns with the objective of analyzing patient-managed medication use within online discourse, safety concerns pertaining to non-oral therapies were not examined and may necessitate separate investigation.

As a methodological limitation, keyword-based data collection possesses inherent constraints, as it depends on predefined search terms to encompass the full semantic spectrum of relevant discussions. Comments that implicitly address oral medication safety, utilize colloquial language, metaphors, or context-specific expressions, may not be captured. Additionally, keyword ambiguity in the Hungarian language may lead to the inclusion of irrelevant content or the omission of pertinent data during data cleaning procedures. It is also pertinent to note that the coding process was carried out by two researchers, which could have influenced the interpretation of categories despite adherence to a predefined framework. To mitigate this, disagreements were resolved through joint review of the framework definitions and consensus-based discussions, thereby supporting analytical consistency while acknowledging the interpretative nature of qualitative coding. Consistent with the interpretive and framework-guided approach of the analysis, formal inter-coder reliability statistics were not computed; consequently, the frequencies reported should be regarded as indicative patterns rather than precise quantitative measures. Moreover, it is important to recognize that negative experiences tend to be expressed more frequently than positive ones within the Hungarian digital space, potentially resulting in the underrepresentation of affirmative care experiences.

While Facebook was one of the primary data source, only publicly accessible content was incorporated. Facebook groups were deliberately excluded, as they do not qualify as public data and raise ethical concerns regarding privacy and informed consent. Consequently, discussions occurring within closed or private groups, as well as on other social media platforms, may exhibit different user populations, norms, or interaction patterns that were not encompassed in this study.

Concerning the patient groups, our knowledge is restricted or nonexistent regarding specific or particular cohorts, due to reliance solely on publicly available data. Information concerning the socioeconomic status of individuals who leave comments remains limited: as for contraceptive medication, it is assumed that the majority of discussions involve women; however, in most regions, it is not possible to ascertain details such as the patient’s age, gender, location, labor market situation, and other relevant demographics. The identity of commenters could not be independently verified, and classification of roles depended on self-disclosure and content-based analysis. The effects of the digital divide may have impacted participation patterns, as older patients and individuals with lower digital literacy are less inclined to engage in online discussions, potentially resulting in the underrepresentation of specific conditions and patient groups.

Children and dental patients were intentionally excluded from the analysis, as medication use, decision-making processes, and safety considerations in these groups follow distinct clinical and social logics compared to the general adult patient population. Including these discussions was therefore considered likely to blur the analytical focus and reduce the conceptual coherence of the study’s scope.

## 5. Conclusions

This study demonstrates that netnography is a suitable and valuable approach for exploring patients’ perspectives on medication safety in the Hungarian context. By analyzing a large volume of publicly accessible online discussions, we show that a previously developed patient-centered medication safety framework is applicable in real-world patient narratives, although unevenly represented across domains.

The findings indicate that patient concerns concentrate primarily on access to healthcare services and communication with healthcare professionals, while other system-level medication safety domains receive less attention in online discourse. Prescription medications—particularly those related to gynecology, internal medicine, and gastroenterology—dominate safety-related discussions, reflecting areas of heightened patient vulnerability and unmet informational needs.

These results suggest that healthcare systems may benefit from greater attention to access-related barriers and patient–professional communication when seeking to support medication safety. Patients appear to turn to online spaces for information and peer support when formal care pathways do not adequately address their concerns, which may increase exposure to misinformation in the absence of professional engagement. Enhancing medication literacy and incorporating patient feedback into organizational learning processes may therefore represent important considerations for patient-centered medication safety strategies.

In addition to its substantive findings, this study highlights the methodological usefulness of netnographic analysis of publicly accessible online discourse as a complementary tool for capturing patient-generated perspectives that are often underrepresented in traditional patient safety research.

## Figures and Tables

**Table 1:** The original framework based on Giles et al. [5].

Issue	Definition
1. Access to services	Access to services that provide prescriptions and medicines, and/or access to health care professionals who can give you information about medicines.
2. Communication	Effectiveness of the exchange and sharing of information about medicines between hospital and general practice, staff, patients, groups, departments, and services.
Communication between HCPs	Lack of effective communication in supplies of medication, changes in dose, formulation.
Communication between healthcare professionals and patients	Lack of appropriate information about medication use, such as medication changes, length of treatment, lack of listening to patient’s concerns about their medication (including medication errors).
3. Computer systems and programs	Failures of systems, poor design, and lack of interfacing between systems.
4. Continuity of care	Continuity of healthcare professionals who deal with medicines (e.g., locum pharmacists and GPs).
5. Dignity and respect	Associated with feeling comfortable, in control and valued.
6. Healthcare professional factors (This tended to focus on the knowledge of HCPs from the patient’s point of view, but also about attitudes.)	Characteristics and knowledge of the person delivering care that may contribute in some way to issues with medicines, e.g., inexperience, stress, personality, attitudes.
7. Medication policies and processes	Policies/directives that impact on the safety of medication usage.
8. Medication safety culture	Organizational values, beliefs, and practices surrounding the management of medication safety and learning from error.
9. Patient- and carer/relative-related factors Patient knowledge Patient responsibility Patient involvement Physical and cognitive	Features of the patient that make involvement in safe use of medicines more difficult and therefore more prone to error (e.g., abnormal physiology, language difficulties, personality). Patient condition affects safety or impact of safety issues.
10. Role and responsibilities	Existence of clear lines of responsibility clarifying accountability of staff members and delineating the job role when dealing with medicines (complaints and lack of clarity around lines of responsibility).
11. Supplies of medication and appliances	Issues surrounding obtaining timely supplies of medicines or appliances.
12. Workload of health care professionals	Perceived level of activity and pressures on time during working hours.

**Table 2:** The distribution of framework categories indicates a pronounced prevalence of issues related to ‘Access to services’ and ‘Communication’ in the online discussions.

Framework Labels	Comments	Percent
Access to services	5123	64.1%
Communication	2580	32.3%
Supplies of medication and appliances	105	1.3%
Healthcare professional factors	54	0.7%
Role and responsibilities	44	0.6%
Medication safety culture	24	0.3%
Medication policies and processes	23	0.3%
Workload of health care professionals	18	0.2%
Continuity of care	13	0.2%
Computer systems and programs	6	0.1%
Patient- and carer/relative-related factors	2	0.0%
Dignity and respect	1	0.0%
Other	1	0.0%
Total ^1^	7994	100%

^1^ Percentages may surpass 100% because individual comments could be allocated to a maximum of two categories when multiple framework-relevant themes were explicitly identified.

**Table 3:** The online discussions were predominantly led by patients, whereas participation from relatives and healthcare professionals was significantly lower.

Commenter	Comments	Percent
Patient	4658	90.0%
Relative	266	5.1%
Not revealed	201	3.9%
Healthcare professional	26	0.5%
Other (sharing other patients’ situations but are not patients themselves)	23	0.4%
Total ^1^	5174	100%

^1^ Comments were categorized into a single primary commenter category.

**Table 4:** Comments concerning prescription (RX) medications clearly predominated over those relating to over-the-counter and herbal products.

Type of Oral Medication	Comments	Percent
RX (medical prescription)	3185	61.6%
OTC (over-the-counter medications)	944	18.2%
Herbal medication	884	17.1%
Not revealed	161	3.1%
Total ^1^	5174	100%

^1^ Comments were categorized into a single primary classification of oral medication.

**Table 5:** Gynaecology and general, non-specialty-specific healthcare domains predominated the online discourse concerning medication safety.

Healthcare Area	Comments	Percent
Gynaecology	1588	28.8%
Unspecified (non-specific symptoms) ^1^	1219	22.1%
Internal medicine	712	12.9%
Gastroenterology	686	12.4%
Neurology	275	5.0%
Cardiology	246	4.5%
Psychiatry	240	4.3%
Allergology	148	2.7%
Endocrinology	118	2.1%
Oncology	46	0.8%
Rheumatology	46	0.8%
Otolaryngology	37	0.7%
Urology	33	0.6%
Dentistry	32	0.6%
Dermatology	22	0.4%
Immunology	12	0.2%
Pulmonology	8	0.1%
Ophthalmology	3	0.1%
Addictology	1	0.0%
Not revealed	48	0.9%
Total ^2^	5520	100%

^1^ Unspecified: non-specific symptoms without sufficient information to assign a specialty. ^2^ Percentages may surpass 100% due to individual comments potentially being classified into up to two categories when multiple themes pertinent to healthcare areas are explicitly identified.

**Table 6:** ATC groups G (genito-urinary system and sex hormones) and A (alimentary tract and metabolism) predominated in the medication-related remarks.

ATC Codes	Comments	Percent
G—genito urinary system and sex hormones	1328	23.5%
A—alimentary tract and metabolism	1158	20.5%
N—nervous system	430	7.6%
J—antiinfectives for systemic use	426	7.5%
C—cardiovascular system	218	3.9%
B—blood and blood forming organs	185	3.3%
R—respiratory system	166	2.9%
M—musculo-skeletal system	164	2.9%
H—systemic hormonal preparations, excl. Sex hormones and insulins	99	1.8%
P—antiparasitic products, insecticides and repellents	66	1.2%
V—various	36	0.6%
L—antineoplastic and immunomodulating agents	27	0.5%
D—dermatologicals	17	0.3%
S—sensory organs	1	0.0%
Not identifiable (ATC) ^1^	1335	23.6%
Total ^2^	5656	100%

^1^ Not identifiable (ATC): no medicine identifiable to allow ATC coding. ^2^ Percentages may exceed 100% because individual comments could be assigned to up to two categories when multiple ATC codes-relevant themes were explicitly present.

## Data Availability

The processed data presented in this study are available on request from the corresponding author due to ethical reasons.

## Abbreviations

The following abbreviations are used in this manuscript:

ATC	Anatomical Therapeutic Chemical (ATC) Classification System
HCP	Healthcare Professional (or Provider/Personnel)
OTC	Over-The-Counter (medications)
RX	Prescription (medications)
WHO	World Health Organization

## Appendix A

### Appendix A.1

In order to ensure the reproducibility of the research, we share the used keywords: _all: ((tabletta* OR kapszula* OR szirup* OR szuszpenzió*) AND NOT (gyerek* OR gyermek* OR turisztika* OR szálloda* OR hotel* OR foci* OR kutya* OR macska* OR cica* OR hörcsög* OR egér* OR patkány* OR állat* OR kávé* OR mosás* OR mosogatógép* OR erőmű* OR bor* OR öblítő* OR főz* OR mosás* OR színésznő* OR színész* OR piercing* OR tetovál* OR kocsi* OR autó* OR szörp* OR kokain* OR heroin* OR ecstasy* OR marihuána* OR kannabisz* OR kamera* OR szelfi* OR mikulás* OR film* OR vállakozó* OR repülő* OR nyereményjáték* OR játssz* OR játék* OR realme* OR xiaomi* OR samsung* OR iphone* OR dohány* OR jövedéki* OR radioaktív OR nukleáris* OR atomtámadás* OR terror* OR háború* OR ukrajna* OR ukrán* OR orosz* OR ausztrál* OR román* OR szlovák* OR francia* OR párizs* OR bali* OR múzeum* OR kultúra* OR kóstol* OR edző* OR fagyi* OR fagylalt* OR rúzs* OR kozmetikum* OR szájfény* OR luxusmárka* OR designer* OR reptér* OR repülőtér* OR rakéta* OR üzemanyag* OR benzin* OR dízel* OR fogyás* OR fürdőruha* OR iszlám* OR vallás* OR keresztény* OR katolikus* OR hadsereg* OR elnök* OR amerika* OR libanon* OR korea*))

### Appendix A.2

**Table A1:** Illustrative examples of the framework adapted from Giles et al. [5], based on relevant comments from our dataset.

Issue	Example
1. Access to services	“It was a public holiday, I was sent away from everywhere, no one would prescribe it.”
2. Communication	“They addressed me informally and spoke to me condescendingly.”
3. Computer systems and programs	“I went there in person. A notice said: please submit requests in writing and drop them into the mailbox. This is healthcare digitalization at its highest level.”
4. Continuity of care	“I was sent away from everywhere, no one took responsibility.”
5. Dignity and respect	“The pharmacist spoke in a humane manner.”
6. Healthcare professional factors (This tended to focus on the knowledge of HCPs from the patient’s point of view, but also about attitudes)	“After ten years it turned out that I should never have taken those medications together.”
7. Medication policies and processes	“The pill is reliable, people just don’t read the instructions.”
8. Medication safety culture	“There should be a supportive environment, not ‘this is how it’s always been.”
9. Patient- and carer/relative-related factors	“The family resisted medication use.”
10. Role and responsibilities	“How is the patient supposed to know what can be taken together?”
11. Supplies of medication and appliances	“It’s not available in pharmacies either, even with a prescription.”
12. Workload of health care professionals	“I didn’t see a doctor, everything was handled by the nurse.”

## References

[1] Macdonald M.T., Heilemann M.V., MacKinnon N.J., Lang A., Gregory D., Gurnham M.E., Fillatre T. (2014). Confirming Delivery: Understanding the Role of the Hospitalized Patient in Medication Administration Safety. Qual. Health Res..

[2] Báldy B., Safadi H., Lám J. (2023). Betegek a betegbiztonságról (Patients about patient safety). IME—Az Egészségügyi Vez. Szaklapja.

[3] WHO (2017). Medication Without Harm. https://www.who.int/initiatives/medication-without-harm.

[4] Sheikh A., Rudan I., Cresswell K., Dhingra-Kumar N., Tan M.L., Häkkinen M.L., Donaldson L. (2019). Agreeing on global research priorities for medication safety: An international prioritisation exercise. J. Glob. Health.

[5] Giles S.J., Lewis P.J., Phipps D.L., Mann F., Avery A.J., Ashcroft D.M. (2020). Capturing Patients’ Perspectives on Medication Safety: The Development of a Patient-Centered Medication Safety Framework. J. Patient Saf..

[6] Walrath J.M., Rose L.E. (2008). The medication administration process—Patients’ perspectives. J. Nurs. Care Qual..

[7] Wheeler C., Furniss D., Galal-Edeen G.H., Blandford A., Franklin B.D. (2020). Patients’ Perspectives on the Quality and Safety of Intravenous Infusions: A Qualitative Study. J. Patient Exp..

[8] Vanwesemael T., Boussery K., Van Den Bemt P., Dilles T. (2018). The willingness and attitude of patients towards self-administration of medication in hospital. Ther. Adv. Drug Saf..

[9] Vanwesemael T., Boussery K., Manias E., Petrovic M., Fraeyman J., Dilles T. (2018). Self-management of medication during hospitalisation: Healthcare providers’ and patients’ perspectives. J. Clin. Nurs..

[10] Jeacle I. (2021). Navigating netnography: A guide for the accounting researcher. Financ. Account. Manag..

[11] Bratucu R., Gheorghe I.R., Radu A., Purcarea V.L. (2014). The relevance of netnography to the harness of Romanian health care electronic word-of-mouth. J. Med. Life.

[12] Anatomical Therapeutic Chemical (ATC) Classification. https://www.who.int/tools/atc-ddd-toolkit/atc-classification.

[13] Ipsos (2022). Ipsos Global Health Service Monitor 2022. https://www.ipsos.com/sites/default/files/ct/news/documents/2022-09/Ipsos-global-health-service-monitor-2022-VDEF.pdf.

[14] Bíró A., Hajdu T., Kertesi G., Prinz D. (2021). Life expectancy inequalities in Hungary over 25 years: The role of avoidable deaths. Popul. Stud..

[15] Partners H. (2024). Egy Lépést Hátra, Kettőt Előre—A Magyar Magánegészségügy a 2023-as Számok Tükrében (One Step Back, Two Steps Forward—Hungarian Private Healthcare in 2023). https://healce.com/mars-hasab/egy-lepest-hatra-kettot-elore-a-magyar-maganegeszsegugy-a-2023-as-szamok-tukreben/.

[16] (2023). SZEBB-Program. https://www.mgyk.hu/a-szebb-program-anyagai1.html.

[17] Bishop A.C., Macdonald M. (2017). Patient Involvement in Patient Safety: A Qualitative Study of Nursing Staff and Patient Perceptions. J. Patient Saf..

[18] Ringdal M., Chaboyer W., Ulin K., Bucknall T., Oxelmark L. (2017). Patient preferences for participation in patient care and safety activities in hospitals. BMC Nurs..

[19] Sahlstrom M., Partanen P., Azimirad M., Selander T., Turunen H. (2019). Patient participation in patient safety—An exploration of promoting factors. J. Nurs. Manag..

[20] Harris K., Softeland E., Moi A.L., Harthug S., Storesund A., Jesuthasan S., Sevdalis N., Haugen A.S. (2020). Patients’ and healthcare workers’ recommendations for a surgical patient safety checklist—A qualitative study. BMC Health Serv. Res..

[21] Christiansen A.B., Simonsen S., Nielsen G.A. (2021). Patients Own Safety Incidents Reports to the Danish Patient Safety Database Possess a Unique but Underused Learning Potential in Patient Safety. J. Patient Saf..

[22] Street M., Dempster J., Berry D., Gray E., Mapes J., Liskaser R., Papageorgiou S., Considine J. (2021). Enhancing active patient participation in nursing handover: A mixed methods study. J. Clin. Nurs..

[23] Rodrigo-Rincon I., Irigoyen-Aristorena I., Tirapu-Leon B., Zaballos-Barcala N., Sarobe-Carricas M., Antelo-Caamaño M., Lobo-Palanco J., Martin-Vizcaino M. (2020). Do Patients and Relatives Have Different Dispositions When Challenging Healthcare Professionals About Patient Safety? Results Before and After an Educational Program. J. Patient Saf..

[24] KSH (2024). 22.1.1.10. Halálozások a Gyakoribb Halálokok és Nem Szerint (Deaths by Most Common Cause and Sex). https://www.ksh.hu/stadat_files/nep/hu/nep0010.html.

